# A case of hereditary angioedema due to C1-inhibitor deficiency with recurrent abdominal pain diagnosed 40 years after the occurrence of the initial symptom

**DOI:** 10.1007/s12328-021-01338-1

**Published:** 2021-02-05

**Authors:** Daisuke Honda, Isao Ohsawa, Keiichi Iwanami, Hisaki Rinno, Yasuhiko Tomino, Yusuke Suzuki

**Affiliations:** 1 Department of Nephrology, Faculty of Medicine, Juntendo University, Tokyo, Japan; 2 Nephrology Unit, Internal Medicine, Saiyu Soka Hospital, 1-7-22 Matsubara, Soka, Saitama 340-0041 Japan; 3 Department of Rheumatology, Tokyo Bay Urayasu Ichikawa Medical Center, Chiba, Japan; 4 Medical Corporation SHOWAKAI, Tokyo, Japan

**Keywords:** Abdominal pain, Bradykinin, C1-inhibitor, Hereditary angioedema, Icatibant

## Abstract

Hereditary angioedema due to C1-inhibitor deficiency (HAE-C1-INH) is a rare disease, which induces an acute attack of angioedema mediated by bradykinin. HAE-C1-INH can cause serious abdominal pain when severe edema develops in the gastrointestinal tract. However, because it takes a long time, 13.8 years on average in Japan, from the occurrence of the initial symptom to the diagnosis due to low awareness of the disease, undiagnosed HAE-C1-INH patients sometimes undergo unnecessary surgical procedures for severe abdominal pain. We herein present a 56-year-old patient with HAE-C1-INH, who underwent numerous abdominal operations. He frequently needed hospitalization with the administration of opioid due to severe abdominal pain. However, after he was accurately diagnosed with HAE-C1-INH at 55 years of age, he could start self-administration for an acute attack with icatibant, a selective bradykinin B2 receptor antagonist. Consequently, he did not need hospitalizing for ten months after the beginning of the treatment. A series of an accurate diagnosis and appropriate treatment for HAE-C1-INH improved his quality of life. Thus, HAE-C1-INH should be considered, when we meet patients with unidentified recurrent abdominal pain. This case highlights significance of an early diagnosis and appropriate treatment for HAE-C1-INH.

## Introduction

Hereditary angioedema due to C1-inhibitor (C1-INH) deficiency (HAE-C1-INH) can produce excess bradykinin in the kallikrein–kinin system, unpredictably and recurrently inducing acute subcutaneous and/or submucosal angioedema [1–3]. Although HAE-C1-INH is a rare autosomal dominant disease, about 25% of patients with HAE-C1-INH are related to de novo mutations. HAE-C1-INH can be life-threatening when severe edema develops in the upper respiratory tract [4, 5]. Acute attacks can be triggered by surgical procedures, mental stress, trauma, drugs, and infection. The initial symptoms of HAE-C1-INH typically occur in childhood [6]. However, it takes 13.8 years on average in Japan from the occurrence of the initial symptom to its diagnosis [7]. Diagnostic delay not only deprives patients of the opportunity to receive appropriate treatment, but it could also lead to unfortunate results; patients with undiagnosed HAE-C1-INH have reportedly undergone unnecessary surgical procedures for severe abdominal pain due to gastrointestinal edema during an acute attack [8, 9]. Thus, early diagnosis of HAE-C1-INH is important by screening blood tests; C1-INH deficiency spontaneously activates the classical pathway in the complement system resulting in low levels of serum C4 and CH50 with a normal serum C3 level in almost all patients with HAE-C1-INH [10]. We herein present a case of HAE-C1-INH, in which a patient was diagnosed 40 years after the onset of the initial symptom.

## Case report

The patient is a 56-year-old male who underwent appendectomy for severe abdominal pain at 15 years of age (Table 1). After the surgery, he intermittently experienced acute abdominal pain approximately once a week, and he repeatedly was admitted to the hospital for several days. Between 33 and 34 years of age, he underwent various surgical procedures 5 times, and he had to remain in the hospital for 9 months due to medically unexplained abdominal pain. He then underwent jejunectomy for intestinal obstruction at 35 years of age, adhesiotomy at 36 and 48 years of age, and jejunum-colic bypass at 50 years of age. Administration of opioids was simultaneously begun for removal of severe abdominal pain from 46 years of age, and he regularly visited a hospital for the treatment. In addition, he experienced upper respiratory tract edema 4 times, at 48, 49, and 53 years of age, and priapism twice, at 30 s and 53 years of age. He needed hospitalization 19 times a year on average for a decade due to severe abdominal pain.

**Table 1:** Timeline in this HAE-C1-INH patient

Age (year old)	Clinical episode	History of surgical procedure	Number of hospitalization (time)
15	Onset of symptom	Appendectomy	
30s		Phlebotomy for priapism	
33–34		5 surgical abdominal operations	
35		Ileus operation, enterectomy	
36		Abdominal adhesiotomy	34
45			8
46	Beginning of taking opioid		19
47			27
48	Upper respiratory tract edema twice	Abdominal adhesiotomy	25
49	Upper respiratory tract edema		41
50		Jejunum-colic bypass	16
51			19
52			9
53	Upper respiratory tract edema	Phlebotomy for priapism	9
54			5
55	Diagnosis of HAE-C1-INH		8
56			0

**Fig. 1:**
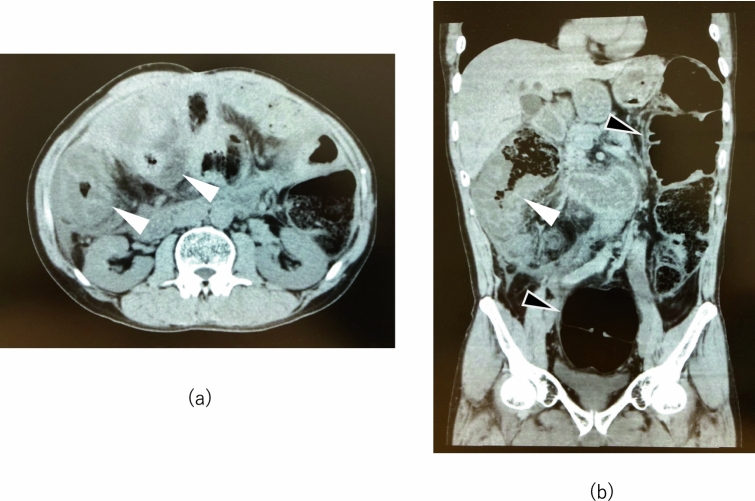
Abdominal computed tomography scan in this HAE-C1-INH patient during an acute attack: **a** axial section and **b** coronal section. Submucosal edema of the intestinal tracts is observed during an acute attack in **a** an axial section and **b** a coronal section (white arrows). Colectasia is observed in **b** a coronal section, which was probably caused by the attenuation of peristalsis due to 11 abdominal surgical procedures (black arrows). HAE-C1-INH, hereditary angioedema due to C1-inhibitor deficiency

**Fig. 2:**
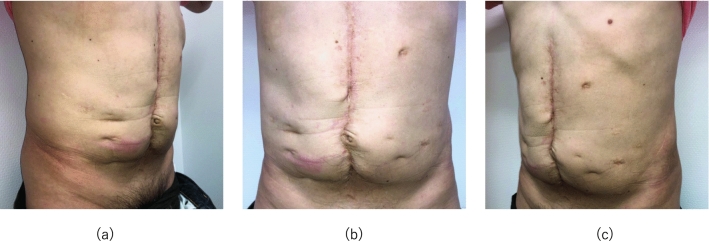
Several abdominal operation scars in this HAE-C1-INH patient **a** right side, **b** center, and **c** left side. The HAE-C1-INH patient has several abdominal operation scars throughout 11 abdominal surgical procedures during the undiagnosed period. HAE-C1-INH, hereditary angioedema due to C1-inhibitor deficiency

At 55 years of age, he consulted a rheumatologist, and the physician noticed low levels of serum C4 (3–16 mg/dl) and CH50 (29 U/ml) with normal serum C3 levels (79–102 mg/dl) as well as characteristic computed tomography (CT) scan findings of submucosal edema of the intestinal tracts and colectasia during an acute attack (Fig. 1). Due to HAE-C1-INH concerns, an additional blood test was performed, revealing extremely low function level of C1-INH (25.2%). An additional medical interview focusing on family history revealed that he repeatedly saw his mother undergo angioedema around the mouth and extremities in his childhood. Accordingly, 40 years after the onset of the initial symptom, he was diagnosed as having HAE-C1-INH. His physician prescribed attenuated androgens for long-term prophylaxis and began administration of plasma-derived human C1-INH concentrate (Berinert P®, CSL Behring, King of Prussia, PA, USA) 1000U for an acute attack, which was effective only for a short period; opioid administration was then restarted for recurrent severe abdominal pain. Subsequently, the physician referred him to us for the management of HAE-C1-INH. He showed numerous surgical scars on the abdomen (Fig. 2). We reconfirmed that the patient had HAE-C1-INH through laboratory examinations (C1-INH function level: < 25.0%), although serum C4 and CH50 levels were elevated to the normal values by the previously prescribed attenuated androgens (C4: 22 mg/dl, CH50: 54 U/ml, C3: 97 mg/dl). We, therefore, began prescription of icatibant (Firazyr®, Takeda Pharmaceutical Company, Tokyo, Japan), a selective bradykinin B2 receptor antagonist, which is available for self-administration during an acute attack. We also prescribed etizolam (Depas®, Mitsubishi Tanabe Pharma Corporation, Osaka, Japan) and suvorexant (Belsomra®, Merck Sharp and Dohme, Kenilworth, NJ, USA), which was intended to reduce attack triggers by stabilizing the patient’s mental state, because the attacks are sometimes caused by the patient’s anxiety. The patient successfully performed icatibant self-administration for acute attacks and was able to lead a normal daily life without hospitalizations for 10 months.

## Discussion

Diagnosis of HAE-C1-INH from the occurrence of the initial symptoms is unacceptably delayed for an average of 8.5 years in the world, and 13.8 years particularly in Japan, mainly due to low awareness of the disease [7, 11]. Furthermore, its symptoms including abdominal pain overlap with those of other forms of angioedema and gastroenterological diseases, which can lead to misdiagnosis [12]. In addition to these factors, because the patient had no family members with confirmed diagnosis of HAE-C1-INH, he might not have chances to be screened for the disease for 40 years after the occurrence of the initial symptom. Retrospectively taking the patient’s history shown in Table 1 and his diagnosis of HAE-C1-INH into consideration, we are convinced that the abdominal pain, upper respiratory tract edema, and priapism during his life were caused by acute attacks due to HAE-C1-INH. As he repeatedly underwent surgical procedures for medically unexplained abdominal pain, his HAE-C1-INH acute attacks became more frequent, probably triggered by the adhesion and the anatomical changes caused by those surgical procedures, as has been previously reported [13]. Thus, this case highlights the importance of an early diagnosis and appropriate treatment through raising awareness of the disease.

HAE-C1-INH is a rare genetic disorder caused by a C1-INH deficiency. Patients with HAE-C1-INH experience their first attack in childhood, and its frequency increases around puberty. The skin, the upper respiratory tract, and the gastrointestinal tract are involved in 98% of attacks; attacks involving abdominal pain are a common manifestation of HAE-C1-INH. Patients with HAE-C1-INH typically have normal routine laboratory values. CT scanning of the abdomen can be useful for detecting gastrointestinal angioedema; however, imaging studies themselves are not specific enough for the diagnosis. If clinical histories or imaging studies are suggestive of HAE-C1-INH, serum complement screening should be undertaken. In patients with HAE-C1-INH, screening typically reveals low C4, low CH50 and normal C3 levels. Confirmed diagnosis of HAE-C1-INH requires a reduced C1-INH function level.

Early treatment by self-administration for an acute attack of HAE-C1-INH prevents its progress to a severe condition, leading to an earlier recovery and shorter painful episodes during attacks [14]. Because the patient had a propensity to endure progressive abdominal pain and visit a hospital in the poorest condition, we persuaded him to change his views on the disease and the treatment strategy for an acute attack, and we trained him to perform self-administration of icatibant as early as possible when an acute attack began, as the guideline of the World Allergy Organization and the European Academy of Allergy and Clinical Immunology recommends [15]. Subsequently, the accurate diagnosis and appropriate treatments for HAE-C1-INH relieved his symptoms and improved his quality of life for 10 months with no hospitalization needed, as shown in Table 1. This improvement was likely due to the medical efficacy of icatibant and the education on early treatment. In addition, we believe that the patient’s knowledge that he can self-administer icatibant at any time and any place can relieve his stress as well as anxiolytic drugs, which could attenuate the occurrence of an acute attack as previously reported [16]. Although the role of trigger factors in the pathophysiology of angioedema has not yet been clarified, providing mental health support or antidepressant treatment should be considered when necessary, because one-third of the edematous episodes which patients experienced resulted from mental stress [17].

On the other hand, because the patient still needs opioids for unendurable abdominal pain despite early treatment by self-administration with icatibant, he has not been completely relieved of symptoms. Although he has depended heavily on removing pain with opioids for a decade, we attempt to reduce the frequency of opioid administration so that his symptoms can be controlled employing only the primary HAE-C1-INH treatment strategy. To achieve this control, we also hope that long-term prophylaxis for HAE-C1-INH will soon be available in Japan. Moreover, increasing awareness of HAE-C1-INH in Japan is urgently necessary. In conclusion, HAE-C1-INH should be considered when patients present with unidentified recurrent serious abdominal pain, and early diagnosis and appropriate treatment for HAE-C1-INH are important.

## Abbreviations

C1-INH: C1-inhibitor
HAE-C1-INH: Hereditary angioedema due to C1-inhibitor deficiency
