# Correction to: A case of hereditary angioedema due to C_1_-inhibitor deficiency with recurrent abdominal pain diagnosed 40 years after the occurrence of the initial symptom

**DOI:** 10.1007/s12328-021-01390-x

**Published:** 2021-03-23

**Authors:** Daisuke Honda, Isao Ohsawa, Keiichi Iwanami, Hisaki Rinno, Yasuhiko Tomino, Yusuke Suzuki

**Affiliations:** 1 Department of Nephrology, Faculty of Medicine, Juntendo University, Tokyo, Japan; 2 Nephrology Unit, Internal Medicine, Saiyu Soka Hospital, 1-7-22 Matsubara, Soka, Saitama 340-0041 Japan; 3 Department of Rheumatology, Tokyo Bay Urayasu Ichikawa Medical Center, Chiba, Japan; 4 Medical Corporation SHOWAKAI, Tokyo, Japan

## Correction to: Clinical Journal of Gastroenterology 10.1007/s12328-021-01338-1

In the original publication of the article, the table 1 was published with a misaligned value under the column head “History of surgical procedure”. The correct Table 1 is published in this correction.

**Table 1:** Timeline in this HAE-C1-INH patient

Age (year old)	Clinical episode	History of surgical procedure	Number of hospitalization (time)
15	Onset of symptom	Appendectomy	–
30s		Phlebotomy for priapism	–
33–34		5 surgical abdominal operations	–
35		Ileus operation, enterectomy	–
36		Abdominal adhesiotomy	34
45			8
46	Beginning of taking opioid		19
47			27
48	Upper respiratory tract edema twice	Abdominal adhesiotomy	25
49	Upper respiratory tract edema		41
50		Jejunum-colic bypass	16
51			19
52			9
53	Upper respiratory tract edema	Phlebotomy for priapism	9
54			5
55	Diagnosis of HAE-C1-INH		8
56			0

*HAE-C1-INH* hereditary angioedema due to C1-inhibitor deficiency

The original article has been corrected.

